# Community pharmacists’ attitudes toward and practice of pharmacy-based harm reduction services in Pittsburgh, PA: a descriptive survey

**DOI:** 10.1186/s12954-024-01018-6

**Published:** 2024-07-19

**Authors:** Caitlin O’Brien, Stephanie Klipp, Raagini Jawa, J. Deanna Wilson

**Affiliations:** 1 School of Medicine, University of Pittsburgh, Pittsburgh, PA USA; 2 Prevention Point, Philadelphia, PA USA; 3 Division of General Internal Medicine, Center for Research in Healthcare, University of Pittsburgh, Pittsburgh, PA USA; 4 Department of Family Medicine and Community Health, University of Pennsylvania, Philadelphia, PA USA

**Keywords:** Harm reduction, Naloxone, Non-prescription needles, Intravenous drug use, Community pharmacy services

## Abstract

**Background:**

In Pittsburgh, PA, legal changes in recent decades have set the stage for an expanded role for community pharmacists to provide harm reduction services, including distributing naloxone and non-prescription syringes (NPS). In the wake of the syndemics of the COVID-19 pandemic and worsening overdose deaths from synthetic opioids, we examine knowledge, attitudes, and practices of harm reduction services among community pharmacists in Pittsburgh and identify potential barriers of expanded pharmacy-based harm reduction services.

**Methods:**

We provided flyers to 83 community pharmacies within a 5-mile radius of the University of Pittsburgh Medical Center to recruit practicing community pharmacists to participate in an anonymous electronic survey. We used a 53-question Qualtrics survey consisting of multiple-choice, 5 or 6 point-Likert scale, and open-ended questions adapted from 5 existing survey instruments. Survey measures included demographics, knowledge, attitudes, and practices of harm reduction services (specifically naloxone and NPS provision), and explored self-reported barriers to future implementation. Data was collected July–August 2022. We conducted descriptive analysis using frequencies and proportions reported for categorical variables as well as means and standard deviations (SD) for continuous variables. We analyzed open-ended responses using inductive content analysis.

**Results:**

Eighty-eight community pharmacists responded to the survey. 90% of participants agreed pharmacists had a role in overdose prevention efforts, and 92% of participants had previously distributed naloxone. Although no pharmacists reported ever refusing to distribute naloxone, only 29% always provided overdose prevention counseling with each naloxone distributed. In contrast, while 87% of participants had positive attitudes toward the usefulness of NPS for reducing disease, only 73% of participants ever distributed NPS, and 54% had refused NPS to a customer. Participants endorsed a lack of time and concerns over clientele who used drugs as the most significant barriers to offering more comprehensive harm reduction services.

**Conclusions:**

Our findings highlight that while most community pharmacists have embraced naloxone provision, pharmacy policies and individual pharmacists continue to limit accessibility of NPS. Future expansion efforts for pharmacy-based harm reduction services should not only address the time and labor constraints identified by community pharmacists, but also fear-based policy and stigma toward people who inject drugs and harm reduction more broadly.

## Introduction

Despite increased public attention, overdose deaths involving synthetic opioids have continued to rise [1]. In light of this, attention has shifted from a traditional emphasis on purely treating substance use disorder with the goal of abstinence to a broader policy agenda supporting the provision of harm reduction services and strategies [2, 3]. Harm reduction is defined as a set of practical strategies and ideas aimed at reducing negative consequences associated with drug use [4]. Research shows that harm reduction practices (such as naloxone and needle and syringe service programs) are effective and cost-effective interventions that decrease incidence of overdose and infection [5, 6]. Despite evidence of their efficacy, there has been limited uptake of harm reduction strategies [7, 8] along with persistent disparities [9]. One potential opportunity to expand harm reduction uptake is to utilize community-based pharmacies. It is estimated that 89% of the US population lives within 5 miles of a pharmacy [10]. Because of the accessibility of outpatient pharmacies, community pharmacists have a unique opportunity to improve health outcomes for patients who use drugs.

In Pittsburgh, PA, current laws set the stage for an expanded role for community pharmacists to provide a broader range of services for people who use drugs. It has been legal to sell needles and syringes without a prescription since 2009, and there has been a statewide standing order for naloxone since 2015. The law allows sales of naloxone and non-prescription syringes (NPS) to occur at the discretion of individual pharmacists. This legal loophole allows pharmacists to act as potential gatekeepers of harm reduction services. For example, in 2018, the CDC found that only one naloxone prescription was dispensed for every 69 high-dose opioid prescriptions, despite the recommendation that they should be prescribed and dispensed together [11]. Secret shopper trials in California (2015), and more recently in Arizona (2023), found that only 21% and 24.6%, respectively, of purchase attempts for nonprescription needles/syringes were successful [12, 13]. Interviews with PWID (persons who inject drugs) revealed that experienced and perceived stigma about getting NPS at pharmacies discourages the use of pharmacies for harm reduction purposes [14]. Many investigations have also found that, even in areas where pharmacists can sell needles and syringes without a prescription, pharmacies have instituted policies limiting access to PWID or any patients without a “legitimate medical reason” [15].

As the need for harm reduction continues to increase, it is important to assess pharmacist participation in and willingness to engage in harm reduction at pharmacies in a legal landscape in which pharmacists have significant control over product distribution. Few studies examine the broad role of pharmacists in offering harm reduction services in the era of synthetic opioids, the COVID-19 pandemic, and rapidly changing drug policy. Our study aims to examine attitudes, practices, and knowledge of harm reduction services broadly among community pharmacists in Pittsburgh and to identify potential barriers and facilitators of expanded harm reduction services.

## Methods

Our survey instrument included questions regarding pharmacist demographics, behaviors, attitudes, and knowledge of harm reduction practices. The 53-item survey included multiple-choice, Likert scale, and open-ended questions adapted from 5 validated survey instruments [16–20]. Pharmacists were then asked to list their frequency of participating in certain harm reduction practices (naloxone distribution, NPS sale, HIV testing, Hepatitis C testing) within their role as a pharmacist. We evaluated attitudes by asking participants to assess their feelings towards people who use drugs and harm reduction practices using 5- and 6-point Likert scale questions (derived from validated survey instruments). We then asked about barriers to expanding implementation of pharmacy-based harm reduction in their practice using a mixture of multiple-choice and short answer questions. We conducted two cognitive interviews to assess question clarity and effectiveness and received feedback on functionality of the online survey from eight student and community member contributors. This study was deemed exempt from human subjects’ research by the University of Pittsburgh Institutional Review Board.

Using an online search, we identified 83 chain, independent, and hospital-associated pharmacies in the greater Pittsburgh area within approximately a 5-mile radius of the University of Pittsburgh Medical Center. We visited each of the identified pharmacies and provided flyers with QR codes linked to the study survey. Participants were eligible if they were a practicing community pharmacist in the Pittsburgh area. Data was collected over 6 weeks (July 2022–August 2022) and participants received $50 compensation for survey completion.

We conducted descriptive analysis using frequencies and proportions reported for categorical variables and means and standard deviations (medians/quartiles for skewed distributions) for continuous variables. Likert scale data on pharmacist attitudes were graphed using diverging bars with split neutrals. Behavior and barrier frequencies were calculated and graphed in bar form. Knowledge questions were scored and reported as percentage correct responses for each of the four questions. Participants responded to two open-ended questions about reasons for refusing to sell NPS and additional barriers to expanding harm reduction services at pharmacies. We analyzed open-ended responses using inductive content analysis.

## Results

A total of 88 community pharmacists participated in the survey. All respondents that completed greater than 10% of the survey questions were included. The number of responses to each survey question varies due to incomplete surveys as well as optional and conditional questions. Differing number of responses for each question are taken into account by reported percentages.

### Demographics

Respondents represented 30 different neighborhoods in Pittsburgh, PA. Participants predominantly identified as white (95.5%; n = 84) and female (64.8%; n = 57), nearly half worked at national chain pharmacies (47.1; n = 41%), and most had worked between 1 and 5 years at their current pharmacy (58.0%; n = 51) (Table 1).

**Table 1:** Survey Participant Demographic and Pharmacy Information

Participant Demographics	Number	%
*Gender (n = 88)*		
Female	57	64.8
Male	31	35.2
*Race/Ethnicity (n = 88)*		
White/Caucasian	84	95.5
Black/African American	1	1.1
Hispanic/Latino	1	1.1
Other	2	2.3
*Age (n = 87)*		
< 25	2	2.3
25–34	44	50.0
35–44	24	27.3
45–54	6	6.8
55–65	9	10.2
> 65	2	2.3
*Pharmacy Type (n = 87)*		
National chain	41	46.6
Regional or local chain	9	10.2
Grocery store pharmacy	17	19.3
Independent	12	13.6
Health-system affiliated outpatient	8	9.1
*Number of years practicing as a pharmacist (n = 88)*		
1–5	31	35.2
6–10	20	22.7
11–20	21	23.9
> 20	16	18.2
*Number of years at current pharmacy (n = 88)*		
1–5	51	58.0
6–10	18	20.5
11–20	10	11.4
> 20	9	10.2

### Legal knowledge

Most pharmacists were very knowledgeable of local laws related to pharmacy-based harm reduction service provision. All (n = 80) respondents correctly identified that Pennsylvania has a Good Samaritan Law allowing citizens to call for emergency assistance for someone who has overdosed without fear of arrest, and most knew that naloxone (95.0%; n = 76) and needles/syringes (97.5%; n = 78) could be sold at pharmacies without a prescription. In comparison, only 76.3% (n = 61) of pharmacists correctly identified that fentanyl test strips can be legally distributed for harm reduction purposes.

**Table 2:** Self-reported Frequency of Harm Reduction Practices by Community Pharmacists in Pittsburgh, Pennsylvania

Question	N	%
*Dispensed Naloxone (n = 87)*		
Yes	80	92.0
No	7	8.0
*Frequency of Naloxone Distribution (n = 80)*		
< 1 time per month	24	30.0
2–3 times per month	32	40.0
Weekly	17	21.3
Daily	7	8.8
*Refused Naloxone (n = 87)*		
Yes	0	0.0
No	87	100.0
*Discuss OD prevention with naloxone sale (n = 80)*		
Never	9	11.3
Sometimes	36	45.0
Often	12	15.0
Always	23	28.8
*Sold non-prescription syringes (n = 85)*		
Yes	62	72.9
No	23	27.1
*Frequency of non-prescription syringe sale (n = 62)*		
< 1 time per month	19	30.6
2–3 times per month	16	25.8
Weekly	9	14.5
Daily	13	21.0
Pharmacy does not sell non-prescription needles	5	8.1
*Refused non-prescription syringes (n = 85)*		
Yes	46	54.1
No	39	45.9
*Discuss safe injection practice with NPS sale (n = 62)*		
Never	38	61.3
Sometimes	16	25.8
Often	3	4.8
Always	1	1.6

### Attitudes

**Fig. 1:**
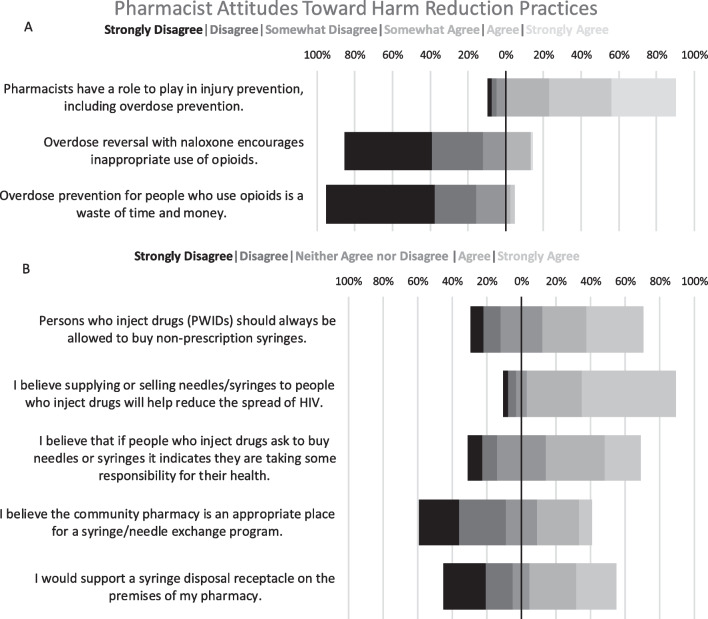
Community Pharmacist Attitudes Toward Pharmacy-based Harm Reduction Practices (n = 82). The percentage of pharmacists that responded to each selection are represented with diverging bar graphs. Panel A contains responses to statements about naloxone and opioid prevention on a 6-point scale from 1 = Strongly disagree to 6 = Strongly agree. Panel B contains responses to statements about non-prescription needles and syringes on a 5-point scale. Responses range from 1 = Strongly disagree to 5 = Strongly agree

We found that 62.7% (n = 52) of participants “somewhat” or “strongly” agreed that illicit opioid use was a problem in their community practice setting, and most felt they possess “a working knowledge of prescription opioid misuse” (95%; n = 76). As shown in Fig. 1, a majority of participants held positive attitudes toward naloxone distribution, agreeing (“strongly agree”, “agree”, and “somewhat agree”) that pharmacists have a role in overdose prevention efforts (90.2%; n = 74). In terms of stigmatizing attitudes, most disagreed (“strongly disagree”, “disagree”, and “somewhat disagree”) that “overdose prevention for people who use opioids is a waste of time and money” (95.1%; n = 78) and that “overdose reversal with naloxone encourages inappropriate use of opioids” (85.4%; n = 70).

Most participants had positive attitudes toward the usefulness of NPS for PWID, “somewhat” or “strongly” agreeing that their sale “helps reduce the spread of HIV” (86.6%; n = 71). A less significant majority agreed (“somewhat” or “strongly”) that PWIDs buying needles or syringes “indicates they are taking some responsibility for their health” (54.9%; n = 45) or that “PWIDs should always be allowed to buy non-prescription needles” (58.9%; n = 48). Respondents mostly disagreed that NPS expansion should occur within community pharmacies, with only 50.0% (n = 41) supporting “a syringe disposal receptacle on the premises of my pharmacy” and only 31.7% (n = 26) endorsing the community pharmacy as an “appropriate place for a syringe/needle exchange program.” Participant responses are illustrated in Fig. 1.

### Harm reduction practices

**Fig. 2:**
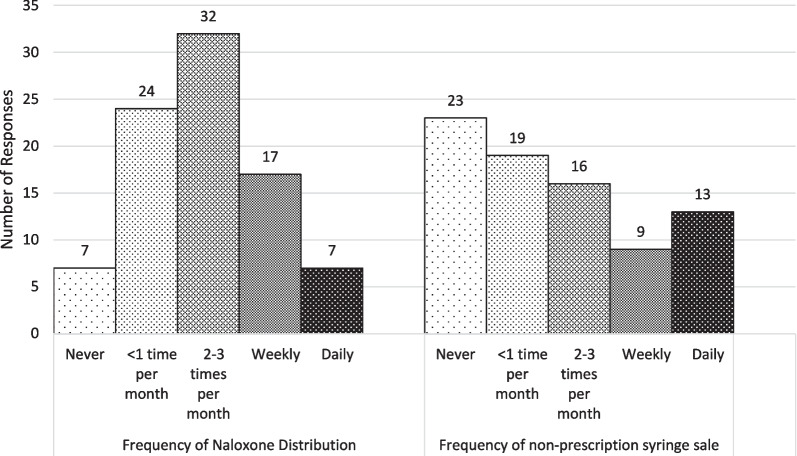
Frequency of Naloxone and Non-Prescription Syringe Distribution by Community Pharmacists in Pittsburgh, Pennsylvania. Participants (n = 85) first responded to a Yes/No question asking if they had ever distributed the product. Those that selected “Yes” responded to an additional question asking them to report their frequency from a multiple choice list (< 1 time per month, 2–3 times per month, Weekly, Daily)

Participant responses regarding the frequency of their participation in harm reduction practices are summarized in Table 2. As seen in Fig. 2, 90.9% (n = 80) of participants had ever distributed naloxone. Among those who distributed naloxone, a majority (70.0%, n = 56) distributed it 3 times or less per month, while a minority (30.0%, n = 24) distributed it weekly or more. No pharmacists reported ever refusing to distribute naloxone. Although the majority were willing to provide naloxone, only 28.8% (n = 23) always provided overdose prevention counseling in conjunction with a naloxone purchase.

In terms of NPS, only 72.9% (n = 62) of participants had ever distributed NPS, and 54.1% had refused to distribute NPS to a customer. Participants’ reasons for refusal fell primarily into three categories: store policies that contradict statewide policies (“All syringe/needle sales must be a prescription.”); concerns for onsite use of NPS (“Due to syringes being found in the parking lot and bathrooms we chose not to sell them due to the risk to other patients.”); and stigmatizing beliefs about NPS as a harm reduction tool (“I do not believe enabling or assisting their behavior is going to help them.”). Of the people who have sold NPS, a majority (61.3%; n = 38) never talk about safe injection practices in conjunction with NPS purchase, and only one pharmacist responded that they always do.

### Implementation barriers

The top barrier cited by participants was “lack of time to develop and implement a harm reduction program”, with 80.0% (n = 64) selecting it as a barrier and 51.3% (n = 41) of pharmacists citing it as their top barrier. The three next most commonly reported barriers were “Complications with billing and reimbursement” (56.2%; n = 45), “Concerns over clientele that might frequent the pharmacy if a program were in place” (51.3%%; n = 41), and “Community opposition to these services” (42.5%; n = 34).

Eight participants described additional barriers. The majority cited having a lack of available staffing and capacity. One participant specified: “I literally don’t have time for more tasks. Pharmacies are at a breaking point because of COVID. I want LESS responsibility”. Another cited concern about proper disposal of distributed harm reduction products.

## Discussion

Our study findings demonstrate community pharmacists in Pittsburgh not only recognize opioid use is common within their local communities but are also aware of recent legal changes supporting an expanded role for pharmacists. While the majority of community pharmacists dispense naloxone and NPS, there is disparity in frequency and comfort between the two interventions, and neither are often paired with harm reduction education.

While Pittsburgh community pharmacists feel that NPS are clinically useful for PWID, there is disagreement whether pharmacies are the appropriate venue for NPS distribution and disposal. This dichotomy between attitudes and behavior is consistent with other literature [16, 17]. A similar proportion of NPS refusals at pharmacies were found by Parry et al. (2021) through a statewide survey of pharmacists in North Carolina [16], suggesting our finding is not an isolated occurrence but a common phenomenon. Similarly, pharmacists expressed less support for disposal of used needles and syringes than provision, mirroring similar survey data from Kentucky [17]. The divergence between acceptance of naloxone compared to NPS may stem from the fact that medication dispensing falls more clearly within the established duties of pharmacists. Anderson et al. found that pharmacists in the UK were more comfortable with providing opioid substitution services than needle exchange programs for this reason [21]. Another explanation could extend to the broader societal acceptance of naloxone compared to NPS. Many states have had standing orders for naloxone for years that allow patients, family, and friends to purchase it at pharmacies without a prescription, and now, as of March 2023, the FDA has approved over-the-counter naloxone nasal spray [22], further increasing accessibility and public comfort. It may also be that needles and syringes are viewed as more central to the stigmatized activity of drug use and refusing to distribute these is a manifestation of stigma. Additional research is needed to identify the causes of this difference and explore how to create effective interventions targeting this disparity.

Our results demonstrated the potential opportunity for the expansion of community pharmacy-based harm reduction to couple distribution of naloxone and NPS with pragmatic education on overdose prevention and safer injection. There is a body of literature that supports that even brief education on naloxone administration in the community setting increases knowledge and confidence in one’s ability to reverse an overdose [23, 24]. Similarly, Phillips et al. showed that a two-session education program on safer injection practices and skin cleaning reduced unclean skin injections among PWID and mitigated infectious complications [25]. Pharmacist-led patient education programs in the inpatient setting have also been shown to increase patient knowledge on naloxone administration [26]. As community pharmacists play an important role in providing education on medications and supplies to manage other disease processes, there is a need to better understand the primary barriers to education provision in the context of these harm reduction strategies.

The majority of pharmacists in our study report limited time and staffing issues as significant barriers to expanding harm reduction services in pharmacies. Since the COVID-19 pandemic, pharmacists have taken on many additional tasks outside the management of prescription drugs [27]. In order to improve access to harm reduction at pharmacies, we need novel strategies that allow pharmacists to offer these services in ways that require a limited investment of staff and time. While previous studies found that knowledge of state laws was a barrier for pharmacists providing harm reduction services [17, 18], pharmacists in our study demonstrated awareness of current laws in Pittsburgh. Additionally, our findings suggest that stigma is still one of the top barriers to broader pharmacy-based harm reduction.

Some participants described restrictive policies concerning NPS within their community pharmacy, limiting NPS distribution. Previous literature has demonstrated that some pharmacies have created policies that require prescriptions or proof of “legitimate” medical use for their sale [15]. Pharmacy-based policies that restrict distribution of NPS further than is required by law need further exploration. Because most respondents correctly identified that NPS are an effective form of harm reduction, the refusal to sell them is likely driven not by a perception of inefficacy but by stigma and harmful assumptions about product use on property, improper needle disposal, and unwanted clientele that would frequent the pharmacy. Pharmacy-based NPS provision has been shown to be effective with minimal associated problems [28]. For example, 70% of New York pharmacists participating in expanded syringe access programs reported experiencing no problems with patients using the program four years after its adoption, despite fears to the contrary at the program onset [29]. We need additional data examining barriers to distribution coupled with targeted education on NPS for pharmacists to dispel fears, correct misbeliefs, and overcome stigma.

There are a few limitations to this study we would like to highlight. Due to the voluntary nature of the survey, the results may selectively capture responses of the pharmacists most interested in the topic of harm reduction, although we attempted to counter this bias with a monetary reward for completing the survey. Given we were sampling from community pharmacists, we are unable to capture the attitudes and practices of pharmacists working in other venues. Additionally, we do not have the ability to know the number of community pharmacists in our study area and are not able to calculate our response rate. Our analysis is limited due to the use of both 5- and 6- point Likert scales. The data on pharmacist behavior is limited to pharmacist perception and self-reported practices and may not accurately reflect practices or behaviors, though we attempted to account for this with anonymity of the survey. Finally, our study may have limited generalizability due to the unique combination of laws, culture and pharmacist education in Pittsburgh.

## Conclusion

Our study shows a majority of community pharmacists in Pittsburgh, PA are participating in harm reduction with variability in acceptance and adoption of naloxone compared to NPS. Few pharmacists couple provision of harm reduction supplies with education on how to use the item to reduce risks (e.g. safer injection practices or overdose prevention), highlighting an area for potential intervention. Furthermore, our findings highlight the key role that stigma against PWID continues to play in reduced uptake of harm reduction services through both interpersonal interactions and structural policies at the level of the pharmacy. Future expansion efforts for pharmacy-based harm reduction services need to address the time and labor constraints identified by community pharmacists, but also explicitly address stigma toward PWID and harm reduction more broadly.

## Data Availability

Deidentified datasets generated during the current study are available from the corresponding author on reasonable request.

## Abbreviations

NPS: Non-prescription syringes
PWID: Persons who inject drugs
HIV: Human immunodeficiency virus
